# Effects of Topical Administration of Bupivacaine HCl to the Ovarian Pedicle on Postoperative Pain, Antioxidant Enzyme Activity and Inflammatory Cytokines in Dogs Undergoing Laparoscopic Ovariectomy

**DOI:** 10.1002/vms3.71144

**Published:** 2026-08-05

**Authors:** Alper Yasin Çiplak, Bülent Polat

**Affiliations:** ^1^ Department of Obstetrics and Gynecology Faculty of Veterinary Medicine Atatürk University Erzurum Turkiye

**Keywords:** bupivacaine HCl, dog, laparoscopic ovariectomy, multimodal analgesia, ovarian pedicle

## Abstract

**Background:**

Laparoscopic ovariectomy (LOE) is a common procedure for elective sterilization in dogs and is associated with less tissue trauma than open techniques. However, manipulation of the ovarian pedicle may still induce nociceptive stimulation and contribute to perioperative inflammatory responses and changes in antioxidant enzyme activity. Topical administration of local anaesthetic to the ovarian pedicle may improve perioperative analgesia in minimally invasive surgery.

**Objectives:**

To evaluate the effects of topical administration of bupivacaine HCl to the ovarian pedicle on postoperative pain, serum antioxidant enzyme activity and systemic pro‐inflammatory cytokines in dogs undergoing LOE.

**Methods:**

Twenty‐four clinically healthy, American Society of Anaesthesiologists (ASA) Class I female dogs were enrolled in a prospective, randomized, controlled trial. The dogs were allocated to receive either a total dose of bupivacaine HCl at 2.2 mg/kg (BPLOVE, *n* = 12), divided between the two ovarian pedicles, or an equivalent volume of isotonic saline (LOVE, *n* = 12), applied using the same topical technique. Standardized anaesthesia and surgical protocols were implemented. Postoperative pain was assessed using the University of Melbourne Pain Scale (UMPS) at 0, 2 and 4 h after surgery. Serum samples collected preoperatively, intraoperatively, and at 0, 2 and 4 h postoperatively were analysed for superoxide dismutase (SOD), catalase (CAT), interleukin‐1β (IL‐1β), interleukin‐6 (IL‐6) and tumour necrosis factor‐α (TNF‐α). Data were analysed using repeated‐measures ANOVA and non‐parametric tests (*p* < 0.05).

**Results:**

All dogs completed the study without experiencing any major complications or requiring intraoperative rescue analgesia. UMPS scores were significantly lower in the BPLOVE group at all postoperative time points (*p* < 0.05). Significant group and time effects were observed in SOD and CAT activities, with higher antioxidant enzyme activity detected in bupivacaine‐treated dogs (*p* < 0.05). Cytokine outcomes showed no consistent between‐group differences; IL‐6 was numerically lower in the BPLOVE group across time points, with a significant difference detected only at 4 h postoperatively.

**Conclusions:**

Topical administration of bupivacaine HCl to the ovarian pedicle during LOE was associated with improved early postoperative analgesia and higher serum SOD and CAT activities, without consistent short‐term changes in systemic pro‐inflammatory cytokine concentrations. These findings support the potential value of ovarian pedicle bupivacaine administration as part of multimodal analgesia in canine laparoscopic surgery and indicate changes in antioxidant enzyme activity during the early perioperative period.

## Graphical abstract

Topical bupivacaine HCl applied to the ovarian pedicle during laparoscopic ovariectomy in dogs significantly reduced early postoperative UMPS pain scores and modulated antioxidant enzyme activity (SOD, CAT). However, systemic pro‐inflammatory cytokine levels (IL‐1β, IL‐6 and TNF‐α) remained unchanged between groups.

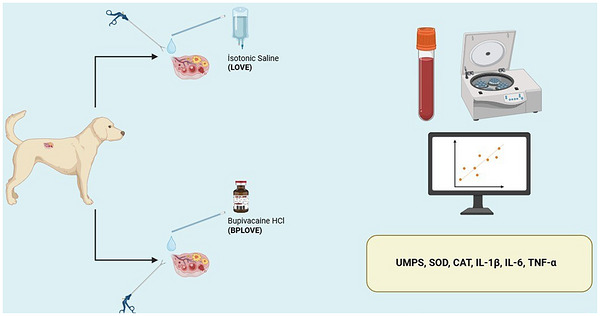

## 1 Introduction

Laparoscopic ovariectomy (LOE) is a minimally invasive surgical technique widely used for elective sterilization in female dogs. Compared with conventional open laparotomy approaches, it is associated with reduced tissue trauma, decreased perioperative pain and faster postoperative recovery, which has contributed to its growing preference in contemporary veterinary practice (Fuertes‐Recuero et al. 2024; Costa et al. 2025; Del Prete et al. 2026). Clinical studies involving LOE combined with minimally invasive gastropexy have also reported acceptable complication rates and mild postoperative pain, further supporting the clinical relevance of laparoscopic techniques in dogs (Leonardi et al. 2020). Despite the minimally invasive character of the procedure, manipulation of the ovarian pedicle, mesovarium and associated ligamentous structures may still elicit clinically relevant nociceptive stimulation during ovariectomy. Previous studies evaluating local anaesthetic techniques directed at the ovarian pedicle or mesovarium support the clinical relevance of this region as a target for perioperative analgesia in dogs undergoing ovariectomy or ovariohysterectomy (Bubalo et al. 2008; Cicirelli et al. 2022). Therefore, the provision of effective perioperative analgesia remains a critical component of LOE from a clinical standpoint.

In the domain of surgical practice, the administration of multimodal analgesic strategies is a widely recommended approach to optimize pain control (Kianian et al. 2024; Graham et al. 2025). The concurrent use of analgesic modalities with divergent mechanisms of action facilitates the suppression of nociceptive transmission at multiple levels, thereby enabling a more stable perioperative course (Yang et al. 2025; Jiang et al. 2025). In this regard, the intraoperative administration of bupivacaine HCl, a long‐acting local anaesthetic, directly to the surgical site has been considered a potentially effective approach. It has been hypothesized that the attenuation of peripheral nociceptive input may serve to limit visceral pain responses and enhance the overall perioperative analgesic efficacy of the procedure (Fuertes‐Recuero et al. 2024; Pezzanite et al. 2023).

Surgical procedures have been shown to induce not only clinical pain symptoms but also a systemic biological stress response characterized by inflammatory activation and changes in antioxidant defence activity. Increases in pro‐inflammatory cytokine levels and changes in antioxidant enzyme activities are considered important indicators of the systemic response to surgery (Savic Vujovic et al. 2023; Zivkovic et al. 2024). The evaluation of these biological parameters in combination with clinical pain scores has the potential to facilitate a more comprehensive understanding of the effectiveness of analgesic approaches.

Despite the existence of literature documenting clinical outcomes associated with LOE (Okur and Polat 2021; Fuertes‐Recuero et al. 2024), investigations examining the impact of local anaesthetic administration to the ovarian pedicle on validated clinical pain scores, serum antioxidant enzyme activity and inflammatory cytokines under controlled clinical conditions remain limited.

The objective of this study was to evaluate the effect of a multimodal analgesia protocol involving the topical administration of bupivacaine HCl to the ovarian pedicle in dogs undergoing LOE on perioperative pain, serum antioxidant enzyme activity and systemic pro‐inflammatory cytokines. The hypothesis of the study was that topical ovarian pedicle bupivacaine administration would reduce postoperative pain and be associated with changes in SOD and CAT activities and circulating pro‐inflammatory cytokine concentrations.

## 2 Materials and Methods

**FIGURE 1:**
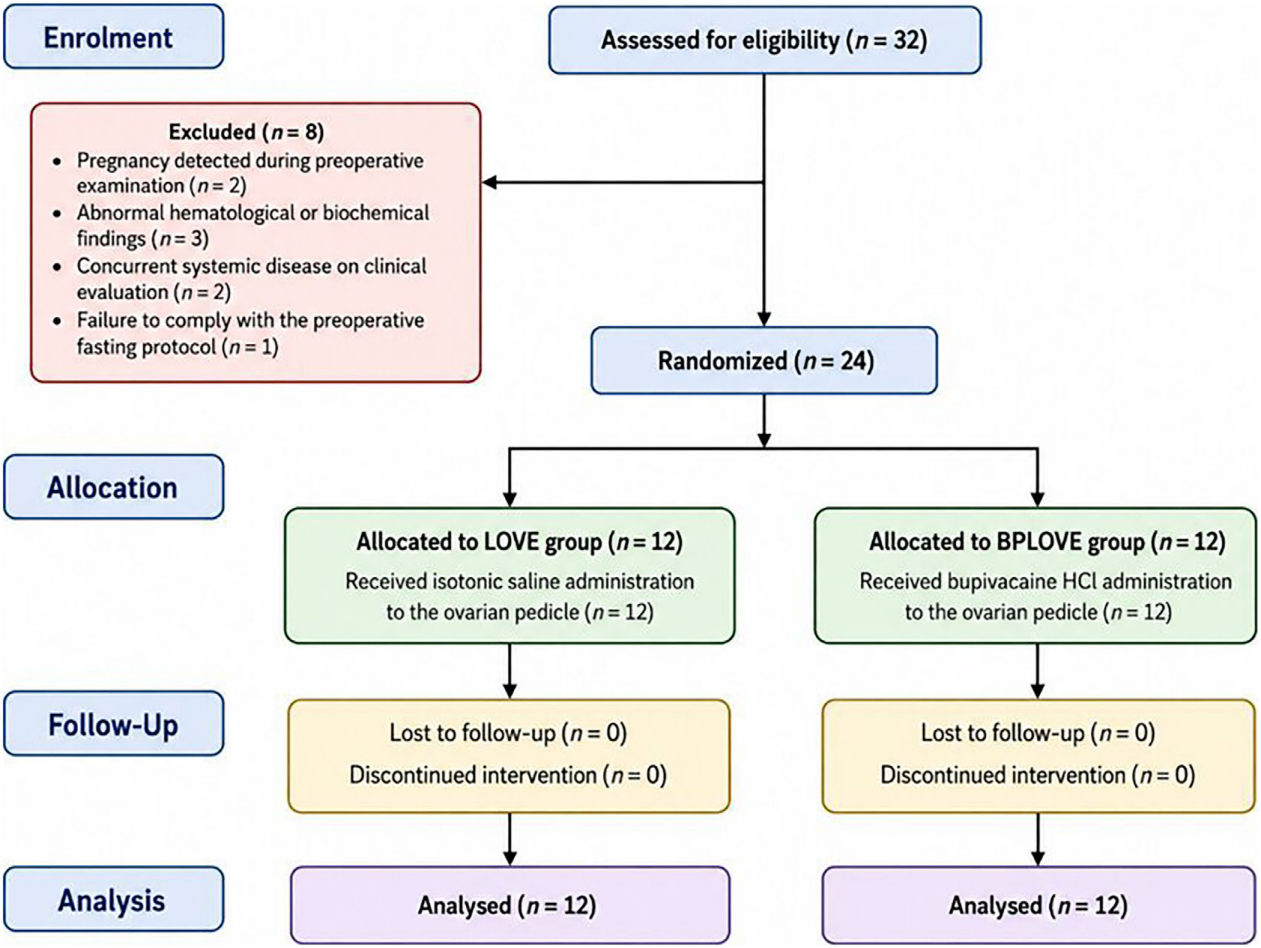
CONSORT flow diagram showing the enrolment, exclusion, randomization, allocation, follow‐up and analysis of dogs included in the study. Of 32 dogs assessed for eligibility, 8 were excluded for predefined clinical reasons, and 24 dogs were randomized equally into the LOVE and BPLOVE groups. All randomized dogs received the allocated intervention, completed follow‐up, and were included in the final analysis.

All animal procedures were conducted in accordance with internationally recognized ethical standards and the European Community Council Directive of 24 November 1986 for the protection of animals used for experimental purposes. The experimental protocol was reviewed and approved by the Atatürk University Animal Experiments Local Ethics Committee (Decision No: 2023/08‐127). The study was designed as a prospective, randomized, controlled clinical trial with blinded outcome assessment and laboratory analyses, and was reported in accordance with the CONSORT 2025 statement for randomized trials (Hopewell et al. 2025) (Figure 1). Throughout the study, perioperative management and monitoring protocols were implemented to reduce surgical stress, nociception and animal discomfort to the greatest possible extent.

### 2.1 Animals

A total of 24 clinically healthy mixed‐breed female dogs, aged 2.1 ± 0.6 years and with a mean body weight of 27.4 ± 3.8 kg, were admitted to the XXX for elective LOE. Before inclusion, all animals underwent a standardized preoperative assessment consisting of a complete physical examination, vaginal cytological evaluation, ultrasonographic examination of the reproductive tract, complete blood count (CBC) and serum biochemical analysis to verify their clinical suitability for surgery. Only dogs classified as American Society of Anaesthesiologists (ASA) physical status I were eligible for inclusion. Oestrous cycle staging was determined based on vaginal cytology, and only dogs not in the follicular phase were included in the study.

Exclusion criteria comprised any evidence of systemic disease, pregnancy or lactation, prior administration of analgesic or anti‐inflammatory medication, abnormal laboratory findings of clinical relevance, or any condition considered to increase anaesthetic or surgical risk. Animals displaying signs of preoperative pain during baseline assessment were also excluded. All dogs were admitted on the day of surgery, subjected to a standardized preoperative fasting protocol, and maintained under routine clinical supervision until induction of anaesthesia. Written informed consent was obtained from the owners of all animals prior to participation.

### 2.2 Groups

Dogs were randomly assigned in a 1:1 ratio using an online randomization software program (www.randomization.org) to one of two groups. The operating surgeons and anaesthetists were not blinded to group allocation. The postoperative pain assessor, laboratory personnel and statistical analyst were blinded to group allocation. Dogs in the BPLOVE group received a total dose of 2.2 mg/kg of bupivacaine HCl (0.5% Buvicaine, Polifarma, Türkiye; equivalent to a total volume of 0.44 mL/kg), which was applied topically to the ovarian pedicles. The total calculated volume was divided equally between the two ovarian pedicles, corresponding to approximately 0.22 mL/kg per pedicle. This volume was selected to provide adequate topical coverage of the ovarian pedicle region under laparoscopic visualization rather than as a free intraperitoneal instillation. The selected dose was based on published canine local and regional anaesthesia literature. In particular, 2.2 mg/kg is identical to the dose reported by Congdon et al. (2017) for psoas compartment and sacral plexus block in dogs and is close to commonly cited clinical doses for local and regional bupivacaine use summarized by Grubb and Lobprise (2020). Dogs in the LOVE group received sterile isotonic saline at the same volume (0.44 mL/kg; NaCl 0.9%, Polifarma, Türkiye), applied to the same anatomical site using the identical technique. The administered volume was individually calculated according to body weight to ensure accurate drug dosing. In the control group, the calculated bupivacaine volume was replaced with sterile saline to maintain consistency of the administered volume between groups.

### 2.3 Anaesthesia

The same anaesthetic protocol was applied to both groups. All dogs were premedicated with 0.2 mg/kg of butorphanol (10 mg/mL, Butomidor, Richter Pharma, Austria) administered subcutaneously (SC) and 0.01 mg/kg of medetomidine (1 mg/mL, Domitor, Orion Pharma, Finland) administered intramuscularly (IM). Approximately 15 min later, the skin over the cephalic vein was aseptically prepared and a peripheral intravenous catheter was inserted to establish vascular access. Lactated Ringer's solution was then administered intravenously (IV) at a rate of 5 mL/kg/h and maintained until the end of anaesthesia. Before anaesthetic induction, dogs were preoxygenated with 100% oxygen via facemask for 3–5 min.

General anaesthesia was induced with 2–4 mg/kg of propofol (20 mg/mL, Propofol‐PF, Polifarma, Türkiye) administered IV, titrated to effect until endotracheal intubation was possible with an appropriately sized cuffed endotracheal tube. Following intubation, the animals were connected to a circle rebreathing anaesthetic system, and anaesthesia was maintained with isoflurane (Forane 250 mL, Aesica Queenborough, England) delivered in 100% oxygen. The vaporized isoflurane concentration was adjusted according to clinical signs to maintain an adequate anaesthetic depth, while the end‐tidal inhalant concentration was maintained between 1.6% and 1.8%. The dogs were allowed to breathe spontaneously throughout the procedure, and ventilatory assistance was provided when necessary to maintain end‐tidal carbon dioxide (EtCO_2_) concentration between 35 and 45 mmHg.

During anaesthesia, physiological variables including heart rate (HR), respiratory rate (*f*R), arterial oxygen saturation (SpO_2_), EtCO_2_ concentration, non‐invasive blood pressure (NIBP) including the systolic (SAP), mean (MAP) and diastolic (DAP) values and rectal temperature (RT) were continuously monitored using a multiparameter monitoring system (Comen, C80‐V, Netherlands). HR was monitored using a three‐lead electrocardiographic system with electrodes placed on the limbs, *f*R was determined by visual assessment of thoracic excursions, SpO_2_ was recorded using a clip‐type pulse oximeter probe positioned on the tongue, EtCO_2_ was monitored using sidestream capnography connected to the endotracheal tube, NIBP was measured using an appropriately sized cuff (width approximately 30%–40% of limb circumference) placed proximal to the carpus, and RT was measured using a rectal probe inserted approximately 5 cm into the rectum.

All anaesthetic procedures were performed under standardized conditions. Dogs were positioned in dorsal recumbency on a heated surgical table and covered with sterile drapes. Active warming was used to maintain body temperature within the physiological range throughout surgery. A single dose of perioperative antibiotic prophylaxis was administered with amoxicillin‐clavulanic acid (Synulox RTU, Zoetis, Italy) at a dose of 8.75 mg/kg SC approximately 30 min before skin incision.

### 2.4 Surgery

All dogs underwent LOE using a three‐port technique. The animals were positioned in dorsal recumbency, and the surgical table was adjusted with a mild tilt, if necessary, to improve visualization of the abdominal organs. To minimize technical variability, all procedures were performed by the same experienced surgeons.

First, a small skin incision was made approximately 2 cm caudal to the umbilicus. A Veress needle was inserted into the abdominal cavity to establish pneumoperitoneum with CO_2_, and intra‐abdominal pressure was maintained within the range of 8–12 mmHg. After insufflation, the Veress needle was removed and a trocar was introduced through the same incision to allow insertion of a 0° laparoscope connected to a video camera system and light source for intraabdominal visualization. Under direct laparoscopic vision, abdominal structures and epigastric vessels were identified to ensure safe placement of the additional working ports.

A second port was placed approximately 5 cm caudal to the primary port, and a third port was positioned a further 4 cm caudal to the second port along the ventral midline under laparoscopic guidance. This configuration was used as a modified three‐port caudal ventral midline approach. The arrangement was selected by the surgical team to provide consistent access to both ovarian pedicles in the study population, facilitate instrument triangulation between the grasping forceps and vessel‐sealing device, and allow controlled placement of the working ports under direct laparoscopic visualization. This approach was not intended to represent the standard three‐port configuration but rather a modified technique used consistently in all dogs to reduce technical variability. The second port was used to insert the vessel‐sealing device (Covidien, LigaSure Maryland Jaw 23 cm, USA). The third port was used to insert grasping forceps (Covidien, Endo Grasp, USA), which were then used to manipulate and exteriorize the ovaries.

Both ovaries were identified and gently grasped with laparoscopic forceps. In dogs allocated to the BPLOVE group, a sterile catheter sheath was introduced through a working port, and bupivacaine HCl solution was applied topically onto each ovarian pedicle under laparoscopic visualization before vascular sealing. The solution was applied slowly to the ovarian pedicle region under direct laparoscopic visualization. No gross leakage or visible pooling away from the target pedicle region was observed during application. After administration of bupivacaine HCl, a 5‐min waiting period was observed for each ovarian pedicle before sealing and transection. This interval was selected based on the reported onset characteristics of bupivacaine in small animal local and regional anaesthesia, with initial onset occurring within approximately 2–5 min and more complete blockade generally developing within 5–10 min (Grubb and Lobprise 2020). In the LOVE group, an equal volume of sterile isotonic saline was applied using the same method, and the procedure then continued according to the standard surgical workflow.

Then, the ovarian pedicles, suspensory ligaments and mesovarium were coagulated and transected using a bipolar vessel‐sealing device, and the ovaries were removed from the abdominal cavity using grasping forceps. After completion of the procedure, the pneumoperitoneum was released via gentle manual abdominal compression, all trocars were removed, and the abdominal incisions were closed in layers using absorbable 2‐0 PGA suture material. The operative time was defined as the elapsed time from the pneumoperitoneum's initiation to the final skin suture.

### 2.5 Postoperative Care

All dogs were monitored during the immediate postoperative period and were discharged once they had fully recovered from anaesthesia and met routine clinical discharge criteria. Postoperative evaluations were performed on Days 3 and 7 following surgery to assess general clinical status, incision healing and the presence of any complications.

Postoperative analgesia consisted of SC administration of meloxicam at a dose of 0.1 mg/kg once daily for two consecutive days, in accordance with standard clinical practice. The first dose was administered after all immediate postoperative pain assessments were completed.

### 2.6 Evaluation of Intraoperative and Postoperative Pain

Intraoperative nociceptive responses to surgical stimulation were monitored using a composite scoring approach derived from Interlandi et al. (2022). A rise of ≥ 20% in HR, *f*R, or SAP relative to baseline values was interpreted as evidence of nociceptive stimulation. Intense surgical stimulation was defined as an increase of ≥ 20% in HR, *f*R or SAP arterial pressure compared with pre‐stimulation baseline values. In addition, marked increases in spontaneous respiratory effort were considered indicative of intraoperative nociception. Whenever these criteria were met, rescue analgesia was provided in the form of intravenous fentanyl at a dose of 2 µg/kg (Fentadon, Dechra, England).

Postoperative pain was evaluated using the UMPS, a validated composite scoring system for pain assessment in dogs. Pain scoring was performed by a single trained observer who was not involved in the surgical or anaesthetic procedures and was blinded to group allocation in order to minimize observer‐related bias. Assessments were conducted at 0, 2 and 4 h after surgery. When the UMPS score exceeded 10/27, rescue analgesia was planned with morphine (0.3 mg/kg IM; Morphine Hydrochloride, Galen İlaç, Türkiye), in accordance with previously published UMPS‐based postoperative pain assessment recommendations and canine ovariectomy studies using comparable rescue analgesia thresholds (Okur and Polat 2021; Hernandez‐Avalos et al. 2019). All dogs were closely monitored during the postoperative assessment period, and rescue analgesia was planned if clinical assessment indicated inadequate analgesia.

### 2.7 Blood Sample Collection

Blood samples were collected at predefined time points consisting of the preoperative period (T_0_), the intraoperative period—immediately after extirpation of the second ovary—(T_1_), and at 0 (T_2_), 2 (T_3_) and 4 (T_4_) h postoperatively. At each sampling, approximately 5 mL of venous blood was drawn from the cephalic vein by the same trained operator in all animals. The cephalic vein was selected because it provided reliable peripheral venous access throughout the perioperative period, allowed repeated sampling with minimal interference with the surgical field and airway management, and enabled a consistent sampling approach across all time points. One portion was placed into K3‐EDTA tubes for haematological analysis (performed at baseline), while the remaining portion was transferred into serum tubes and allowed to clot for subsequent biochemical analyses of antioxidant enzyme activity and inflammatory cytokines.

After collection, the serum tubes were centrifuged within 3–4 h at 1500 × *g* for 15 min to obtain clarified serum. The separated serum was subsequently used for the determination of biochemical parameters related to antioxidant enzyme activity and inflammatory response.

### 2.8 Assessment of Haematological Parameters, Antioxidant Enzyme Activity and Inflammatory Cytokines

A baseline haematological assessment was performed using a CBC obtained prior to anaesthesia induction and analysed with automated haematology techniques. For haematological evaluation, blood samples were collected into EDTA tubes. For biochemical and cytokine analyses, additional blood samples were collected into serum separator vacuum tubes containing clot activator and gel. After clot formation, samples were centrifuged at 1500 × *g* for 15 min to obtain serum. The separated serum samples were stored at −20°C until laboratory analysis.

The study aimed to characterize the biological response to surgical intervention, with particular attention to antioxidant enzyme activity and inflammatory cytokine concentrations. Antioxidant enzyme activity was evaluated by determining the activities of SOD and CAT. The inflammatory response was assessed by measuring serum concentrations of the pro‐inflammatory cytokines IL‐1β, IL‐6 and TNF‐α.

Serum concentrations of canine TNF‐α (Cat No: E0025Ca), IL‐1β (Cat No: E0002Ca) and IL‐6 (Cat No: E0004Ca) were determined using commercially available canine‐specific enzyme‐linked immunosorbent assay (ELISA) kits (Bioassay Technology Laboratory, Shanghai, China) following to the manufacturer's instructions. All samples were analysed in duplicate to ensure assay reliability. Optical densities were read at 450 nm using a microplate ELISA reader (BioTek Instruments, USA), and cytokine concentrations were calculated from standard calibration curves generated for each assay. According to the manufacturer's data sheets, the validation parameters for the cytokine ELISA kits were as follows: for IL‐6, the intra‐assay CV was < 8%, the inter‐assay CV was < 10%, the LOD was 0.02 pg/mL and the LOQ was 0.05–15 pg/mL; for TNF‐α, the intra‐assay CV was < 8%, the inter‐assay CV was < 10%, the LOD was 0.01 pg/mL and the LOQ was 0.03–9 pg/mL; and for IL‐1β, the intra‐assay CV was < 8%, the inter‐assay CV was < 10%, the LOD was 0.1 pg/mL and the LOQ was 0.2–60 pg/mL.

SOD activity was measured using the method proposed by Sun et al. (1988). CAT activity was calculated using the Goth method, which quantifies the amount of hydrogen peroxide decomposed per unit time (Goth 1991). A BioTek Epoch UV‐visible EIA Spectrophotometer (BioTek Instruments, USA) was used to measure the levels of serum SOD and CAT activities. SOD and CAT activities were measured in serum to evaluate circulating antioxidant enzyme activity during the early perioperative period. The same sample matrix and analytical procedures were used for all animals and time points. Although SOD and CAT are predominantly intracellular enzymes and are often measured in erythrocyte hemolysates, serum‐based assessment of SOD and CAT activity has also been used in canine oxidative and antioxidant status studies. Therefore, the present findings should be interpreted specifically as serum antioxidant enzyme activities rather than as direct measures of erythrocyte antioxidant capacity.

### 2.9 Statistical Analysis

A priori sample size estimation was performed using G*Power software (version 3.1.9.7). The calculation was performed for the primary clinical outcome, postoperative UMPS score, using the test family ‘*t*‐tests’ and the statistical test ‘means: difference between two independent means (two groups).’ A two‐tailed analysis was selected with an α error probability of 0.05, a desired power of 0.95, an allocation ratio of 1:1 and an assumed large effect size of Cohen's *d* = 1.54. Because the calculation was based on a standardized effect size, a separate standard deviation (SD) value was not entered into the final G*Power model. The analysis indicated that 12 dogs per group, corresponding to a total sample size of 24 dogs, were required. Since this sample size calculation was based on the postoperative pain outcome rather than serum cytokine concentrations, cytokine analyses were considered exploratory and the possibility of Type II error was acknowledged when interpreting non‐significant cytokine findings. To improve interpretation beyond *p* values, time point‐specific mean differences with 95% confidence intervals and Hedges’ *g* effect sizes were calculated for between‐group cytokine comparisons.

The normality of the data was assessed using the Shapiro–Wilk test. Normally distributed continuous variables are presented as mean ± SD, whereas non‐parametric and ordinal data are presented as median (Q1–Q3). Baseline demographic and surgical characteristics, including age, body weight and surgical time, were compared between the LOVE and BPLOVE groups using the independent‐samples *t*‐test after assessment of normality.

Serum SOD, CAT, TNF‐α, IL‐6 and IL‐1β values were analysed using repeated‐measures analysis of variance (RM‐ANOVA) to evaluate the effects of group, time and group × time interaction. When significant main effects or interactions were detected, pairwise comparisons were performed to assess between‐group differences at the same time point and within‐group changes over time. UMPS scores did not meet the assumptions of parametric testing and were therefore analysed using non‐parametric methods. UMPS scores were presented as median (Q1–Q3), and intergroup comparisons at each postoperative time point were performed using the Mann–Whitney *U* test.

Spearman's rank correlation analysis was used to evaluate time‐matched relationships between UMPS scores and biochemical markers, including SOD, CAT, IL‐1β, IL‐6 and TNF‐α, at 0, 2 and 4 h postoperatively. Statistical significance was set at *p* < 0.05. All analyses were performed using MedCalc statistical software (version 23.2.1, MedCalc Software Ltd., Belgium). Statistical analysis was performed using coded group labels, and the analyst remained blinded to group allocation until all analyses had been completed.

## 3 Results

A total of 32 dogs were initially assessed for eligibility. Eight animals were excluded prior to enrolment for predefined clinical reasons, including detection of pregnancy during preoperative examination (*n* = 2), abnormal haematological or biochemical findings (*n* = 3), evidence of concurrent systemic disease on clinical evaluation (*n* = 2), and failure to comply with the preoperative fasting protocol (*n* = 1).

The remaining 24 dogs met all inclusion criteria and were enrolled in the study. All enrolled dogs completed the study and were included in the statistical analysis. No intraoperative conversion or major perioperative complications occurred. All LOE procedures were completed as planned, and no dog met the predefined criteria for intraoperative rescue analgesia with fentanyl. Baseline demographic and surgical characteristics are summarized in Table 1. Age and body weight did not differ significantly between the LOVE and BPLOVE groups (*p* > 0.05), indicating that the groups were comparable in terms of baseline demographic characteristics. Surgical time was significantly longer in the BPLOVE group than in the LOVE group (*p* = 0.0034), which was mainly attributable to the standardized 5‐min waiting period after topical bupivacaine administration before ovarian pedicle sealing and transection.

**TABLE 1:** Baseline demographic and surgical characteristics of dogs undergoing laparoscopic ovariectomy in the LOVE and BPLOVE groups.

Variable	LOVE (*n* = 12)	BPLOVE (*n* = 12)	*p* value
Age (years)	2.0 ± 0.5	2.2 ± 0.7	> 0.05
Body weight (kg)	27.1 ± 4.0	27.7 ± 3.7	> 0.05
Surgical time (min)	27.4 ± 9.3	40.3 ± 9.8	0.0034

*Note*: Data are presented as mean ± standard deviation. Between‐group comparisons were performed using the independent‐samples *t*‐test after assessment of normality. Surgical time was defined as the elapsed time from initiation of pneumoperitoneum to final skin suture. A *p* value < 0.05 was considered statistically significant.

Abbreviations: BPLOVE: bupivacaine‐treated laparoscopic ovariectomy group; LOVE: laparoscopic ovariectomy group.

### 3.1 Changes in Serum Antioxidant Enzyme Activities

Serum SOD activity showed significant variation during the study period. The overall analysis revealed a significant group effect, with SOD activity being consistently higher in the BPLOVE group than in the LOVE group at most time points (*p* < 0.001). Pairwise comparisons demonstrated that BPLOVE had significantly higher SOD activity than LOVE at T_0_, T_1_, T_3_ and T_4_ (*p* < 0.05), whereas no significant intergroup difference was observed at T_2_. A significant linear effect of time was also detected (*p* < 0.001), indicating temporal changes in SOD activity. In addition, the quadratic time × group interaction was significant (*p* = 0.029), showing that the pattern of change over time differed between the two groups. Within‐group comparisons indicated that SOD activity in the BPLOVE group decreased significantly at T_1_ compared with T0 (*p* < 0.05), while no significant T0‐related temporal change was observed in the LOVE group.

Serum CAT activity also differed between the experimental groups, with a significant group effect being observed (*p* < 0.05). Pairwise comparisons showed no significant difference between LOVE and BPLOVE at T0; however, CAT activity was significantly lower in the BPLOVE group at T_1_ and significantly higher in the BPLOVE group at T_2_, T_3_ and T_4_ compared with the LOVE group at the same time points (*p* < 0.05). A significant linear time effect was identified (*p* < 0.001). Moreover, the linear time × group interaction was highly significant (*p* < 0.001). Within‐group analysis showed that CAT activity increased significantly at T_4_ compared with T0 in the LOVE group (*p* < 0.05). In the BPLOVE group, CAT activity was significantly higher at T_3_ and T_4_ compared with T_0_ (*p* < 0.05, Table 2.).

**TABLE 2:** Effects of ovarian pedicle bupivacaine administration (BPLOVE) versus standard laparoscopic ovariectomy (LOVE) on serum superoxide dismutase (SOD) and catalase (CAT) activities at specific perioperative time points in dogs.

Antioxidant enzymes	Group	Time				
		T_0_	T_1_	T_2_	T_3_	T_4_
SOD (U/mg protein)	LOVE	21 ± 0.23 ^a^	20 ± 0.25 ^a^	20.6 ± 0.22	21 ± 0.18 ^a^	21.5 ± 0.18 ^a^
	BPLOVE	22.3 ± 0.23^b^	21.8 ± 0.25 ^b*^	22.4 ± 0.22	22.9 ± 0.18 ^b^	23.2 ± 0.18 ^b^
CAT (U/mg protein)	LOVE	119 ± 1.8	113.3 ± 1.49^a^	117.9 ± 3.37^a^	122.6 ± 3.59^a^	128.9 ± 3.84^a*^
	BPLOVE	116.2 ± 1.8	105.1 ± 1.49 ^b^	125.9 ± 3.37 ^b^	141.7 ± 3.59^b*^	157.6 ± 3.84^b*^

*Note*: T_0_, preoperative baseline before anaesthesia induction; T_1_, intraoperative, immediately after removal of the second ovary; T_2_, immediately after surgery (0 h); T_3_, 2 h after surgery; T_4_, 4 h after surgery. Data are expressed as mean ± standard deviation (SD). Different lowercase superscript letters within the same time point indicate statistically significant between‐group differences (*p* < 0.05). Asterisks indicate statistically significant within‐group differences compared with T0 (*p < 0.05).

Abbreviations: BPLOVE, bupivacaine‐treated laparoscopic ovariectomy group; CAT, catalase; LOVE, laparoscopic ovariectomy group; SOD, superoxide dismutase.

### 3.2 Changes in Serum Pro‐Inflammatory Cytokine Levels

Serum IL‐1β concentrations did not show a significant difference between the LOVE and BPLOVE groups throughout the study period (*p* > 0.05). Although IL‐1β values showed minor numerical fluctuations over time in both groups, no significant time effect was detected (*p* > 0.05). In addition, the quadratic time × group interaction was not statistically significant (*p* = 0.201), indicating that the temporal pattern of IL‐1β concentrations was comparable between the two groups. Pairwise comparisons also revealed no significant intergroup differences at any individual time point and no significant within‐group changes compared with T0 (*p* > 0.05).

Serum IL‐6 concentrations were numerically lower in the BPLOVE group than in the LOVE group at all evaluated time points. No significant overall group effect, time effect, or time × group interaction was identified (*p* > 0.05). However, pairwise comparisons demonstrated a significant between‐group difference at T_4_, with lower IL‐6 concentrations in the BPLOVE group compared with the LOVE group (*p* = 0.026), whereas no significant intergroup differences were detected at the other time points. No significant within‐group changes from T0 were observed in either group (*p* > 0.05).

Serum TNF‐α concentrations remained statistically comparable between the LOVE and BPLOVE groups during the entire observation period. No significant main effect of group, time, or time × group interaction was detected for TNF‐α levels (*p* > 0.05). Moreover, pairwise comparisons showed no significant intergroup differences at any time point and no significant within‐group alterations relative to T_0_ (*p* > 0.05, Table 3).

**TABLE 3:** Effects of ovarian pedicle bupivacaine administration (BPLOVE) versus standard laparoscopic ovariectomy (LOVE) on serum pro‐inflammatory cytokine concentrations, mean differences, 95% confidence intervals and effect sizes at specific perioperative time points in dogs.

Pro‐inflammatory cytokine	Time	LOVE mean ± SD	BPLOVE mean ± SD	Mean difference	95% CI	*p* value	Effect size, Hedges’ *g*
IL‐1β (pg/mL)	T0	1.89 ± 1.78	1.55 ± 2.35	−0.34	−2.15 to 1.46	0.696	−0.16
IL‐1β (pg/mL)	T1	1.50 ± 1.17	1.96 ± 4.08	0.46	−2.20 to 3.11	0.716	0.14
IL‐1β (pg/mL)	T2	1.45 ± 1.03	2.34 ± 5.17	0.89	−2.43 to 4.21	0.570	0.23
IL‐1β (pg/mL)	T3	1.72 ± 1.38	2.81 ± 7.14	1.08	−3.50 to 5.67	0.616	0.20
IL‐1β (pg/mL)	T4	2.13 ± 2.13	1.59 ± 2.41	−0.55	−2.51 to 1.42	0.570	−0.23
IL‐6 (pg/mL)	T0	0.30 ± 0.24	0.18 ± 0.12	−0.12	−0.29 to 0.06	0.173	−0.59
IL‐6 (pg/mL)	T1	0.27 ± 0.15	0.18 ± 0.14	−0.08	−0.21 to 0.05	0.198	−0.55
IL‐6 (pg/mL)	T2	0.28 ± 0.16	0.17 ± 0.10	−0.12	−0.24 to 0.00	0.057	−0.84
IL‐6 (pg/mL)	T3	0.27 ± 0.21	0.16 ± 0.08	−0.12	−0.27 to 0.03	0.117	−0.69
IL‐6 (pg/mL)	T4	0.29 ± 0.15	0.16 ± 0.09	−0.13	−0.24 to −0.02	0.026	−1.00
TNF‐α (pg/mL)	T0	1.13 ± 0.36	1.05 ± 0.25	−0.09	−0.36 to 0.18	0.496	−0.27
TNF‐α (pg/mL)	T1	1.02 ± 0.10	1.08 ± 0.43	0.06	−0.23 to 0.35	0.651	0.20
TNF‐α (pg/mL)	T2	1.10 ± 0.26	1.01 ± 0.34	−0.09	−0.35 to 0.18	0.491	−0.29
TNF‐α (pg/mL)	T3	1.04 ± 0.25	1.13 ± 0.32	0.09	−0.15 to 0.34	0.438	0.32
TNF‐α (pg/mL)	T4	1.07 ± 0.24	1.04 ± 0.20	−0.03	−0.22 to 0.16	0.735	−0.14

*Note*: T0, preoperative baseline before anaesthesia induction; T1, intraoperative, immediately after removal of the second ovary; T2, immediately after surgery; T3, 2 h after surgery; T4, 4 h after surgery. Data are expressed as mean ± standard deviation. Mean difference was calculated as BPLOVE − LOVE. Effect size was calculated as Hedges’ *g* for between‐group comparisons at each time point. Negative mean differences and negative effect sizes indicate lower concentrations in the BPLOVE group compared with the LOVE group.

Abbreviations: CI, confidence interval; IL‐1β, interleukin‐1β; IL‐6, interleukin‐6; TNF‐α, tumour necrosis factor‐α.

### 3.3 University of Melbourne Pain Scale Scores

UMPS scores revealed significant differences between groups at 0, 2 and 4 h postoperatively. Values in the BPLOVE group were consistently lower than those in the LOVE group at all evaluated postoperative time points (*p* < 0.05). UMPS values showed a progressive decline in both groups over time, indicating a significant reduction in postoperative pain scores throughout the observation period (*p* < 0.001). Despite this time‐dependent decrease, the BPLOVE group had lower pain scores than the LOVE group at the evaluated time points. UMPS data are presented as median (Q1–Q3). Statistically significant intergroup differences at the corresponding postoperative time points are indicated in Table 4. The highest UMPS score recorded in any dog during the 4‐h postoperative assessment period was 5/27. Therefore, no dog reached the predefined rescue analgesia threshold, and no postoperative rescue morphine was required during this period.

**TABLE 4:** Effects of ovarian pedicle bupivacaine administration (BPLOVE) versus standard laparoscopic ovariectomy (LOVE) on postoperative University of Melbourne Pain Scale (UMPS) scores at 0, 2 and 4 h in dogs.

Time	Group	Median (Q1–Q3)	*p* (between groups)
0 h	BPLOVE	1 (1–2)^a^	0.001
	LOVE	3 (3–4)^b^	
2 h	BPLOVE	1 (0–1)^a^	0.013
	LOVE	1 (1–2)^b^	
4 h	BPLOVE	0 (0–0)^a^	0.011
	LOVE	1 (0–1)^b^	

*Note*: UMPS scores are presented as median (Q1–Q3). Between‐group comparisons at each postoperative time point were performed using the Mann–Whitney *U* test. Different superscript letters within the same time point indicate a statistically significant between‐group difference (*p* < 0.05). 0 h, immediately after completion of surgery; 2 h, 2 h postoperatively; 4 h, 4 h postoperatively.

Median UMPS scores decreased over time in both groups. Spearman correlation analysis showed negative correlations between UMPS scores and SOD activity at 0, 2 and 4 h postoperatively. CAT activity was negatively correlated with UMPS scores at 4 h, whereas no consistent significant correlations were detected between UMPS scores and IL‐1β, IL‐6 or TNF‐α concentrations (Table 5).

**TABLE 5:** Spearman correlation analysis between postoperative University of Melbourne Pain Scale (UMPS) scores and biochemical markers at 0, 2 and 4 h after surgery.

Time	Marker	*n*	Spearman's *ρ*	*p* value
0 h	SOD	24	−0.812	< 0.001
0 h	CAT	24	−0.397	0.055
0 h	IL‐1β	23	0.317	0.141
0 h	IL‐6	24	0.305	0.147
0 h	TNF‐α	23	−0.121	0.584
2 h	SOD	24	−0.458	0.024
2 h	CAT	24	−0.241	0.256
2 h	IL‐1β	23	0.336	0.117
2 h	IL‐6	24	0.214	0.316
2 h	TNF‐α	23	0.079	0.718
4 h	SOD	24	−0.485	0.016
4 h	CAT	24	−0.472	0.020
4 h	IL‐1β	23	0.379	0.075
4 h	IL‐6	23	0.317	0.141
4 h	TNF‐α	22	−0.315	0.153

*Note*: Spearman's rank correlation analysis was performed between time‐matched UMPS scores and biochemical markers at 0, 2 and 4 h postoperatively. Missing or non‐evaluable values were excluded pairwise from the corresponding correlation analysis.

Abbreviations: CAT, catalase; IL‐1β, interleukin‐1β; IL‐6, interleukin‐6; SOD, superoxide dismutase; TNF‐α, tumour necrosis factor‐α.

## 4 Discussion

The present randomized clinical study examined the effects of administering topical bupivacaine to the ovarian pedicle on postoperative pain, serum antioxidant enzyme activity and circulating pro‐inflammatory cytokines in female dogs undergoing LOE. The findings indicate that dogs receiving ovarian pedicle bupivacaine had lower early postoperative UMPS scores and higher serum antioxidant enzyme activities (SOD and CAT) than control dogs. Concurrently, systemic cytokine concentrations (IL‐1β, IL‐6 and TNF‐α) remained comparable between treatment groups. Taken together, these observations suggest that ovarian pedicle‐focused analgesia was associated primarily with improved early postoperative pain scores and altered antioxidant enzyme activity, without measurable short‐term differences in circulating pro‐inflammatory cytokine concentrations.

Although LOE is considered minimally invasive, nociceptive stimulation is still clinically significant due to visceral traction, vascular sealing and ligament manipulation when handling the ovarian pedicle. A body of research has demonstrated that the manipulation of ovarian structures during surgical procedures contributes to intraoperative nociceptive input and postoperative discomfort in female dog sterilization operations (Espadas‐González et al. 2022; Degani et al. 2023; Fuertes‐Recuero et al. 2024). Consequently, the ovarian pedicle emerges as a rational target for local analgesic intervention, aimed at disrupting peripheral nociceptive transmission at its origin.

In the present study, animals that received ovarian pedicle bupivacaine exhibited lower UMPS scores during the early postoperative period, suggesting enhanced clinical analgesia. Local anaesthetics, such as bupivacaine, act by reversibly blocking voltage‐gated sodium channels. This prevents the propagation of peripheral nociceptive impulses and limits central sensitization (Kukreja et al. 2022). When delivered directly to tissues subjected to surgical manipulation, this mechanism can substantially reduce visceral afferent signalling. Previous veterinary studies evaluating regional or local anaesthetic techniques during ovariectomy have also reported better postoperative pain management with targeted local anaesthetic application (Paolini et al. 2022; Cavaco et al. 2022). Therefore, the present results reinforce the concept that ovarian pedicle‐specific analgesia is an effective component of multimodal perioperative pain management in female dog minimally invasive sterilization.

Beyond the behavioural outcomes related to pain, a notable observation was the significantly higher SOD and CAT activities detected in the treated animals. Surgical injury has been associated with increased reactive oxygen species production through tissue trauma, inflammatory activation and perioperative metabolic stress (Dragoescu et al. 2025; Cuevas‐Budhart et al. 2025). In dogs undergoing ovariectomy under general anaesthesia, Bruschetta et al. (2024) also reported redox‐related changes associated with ovariectomy, further supporting the relevance of evaluating antioxidant enzyme responses in this surgical model. More recently, Azizi et al. (2025) examined oxidative stress after LOE in dogs using a broader biomarker panel, including total oxidant status (TOS), total antioxidant capacity, malondialdehyde, SOD, CAT and glutathione peroxidase. Their study highlights that interpretation of postoperative oxidative status requires simultaneous assessment of oxidant burden, oxidative damage and antioxidant enzyme activity. In the present study, the higher SOD and CAT activities observed in the BPLOVE group should therefore be interpreted specifically as increased serum antioxidant enzyme activities during the early perioperative period. These findings should be limited to increased serum SOD and CAT activities and should not be extended to broader conclusions regarding oxidative status. Direct oxidative damage markers, such as MDA, protein carbonyls, or 8‐OHdG and global oxidant status parameters such as TOS would be required to determine whether the observed enzyme changes reflect altered oxidant burden, compensatory antioxidant enzyme activation or both.

In contrast to the observed differences in pain scores and antioxidant enzyme activities, circulating pro‐inflammatory cytokines, including IL‐1β, IL‐6 and TNF‐α, did not differ significantly between groups. This finding may be explained by the relatively limited tissue trauma associated with LOE, which is generally associated with lower systemic inflammatory activation compared with open abdominal procedures. Comparative surgical studies have demonstrated that systemic inflammatory responses are closely related to the magnitude of tissue injury and are typically lower following laparoscopic procedures compared with open techniques (Calabria et al. 2026; Naghavi et al. 2025). Furthermore, cytokine responses are highly time‐dependent, and the early postoperative sampling window used in the present study may not have coincided with the expected peak systemic release of IL‐1β, IL‐6 or TNF‐α, which may occur later than the 4‐h observation period. Therefore, the absence of significant intergroup differences should not be interpreted as definitive evidence that inflammatory pathways were unaffected. Rather, these findings indicate that ovarian pedicle bupivacaine administration did not produce a detectable short‐term difference in circulating pro‐inflammatory cytokine concentrations within the selected sampling period. In addition, postoperative meloxicam, although ethically appropriate as part of standard clinical care, may have attenuated systemic inflammatory mediator release during the later postoperative period and thereby reduced the ability to detect subtle differences in circulating cytokine concentrations between groups.

Overall, the findings support the clinical relevance of targeting the ovarian pedicle as a perioperative analgesic target during LOE. Within the early postoperative window evaluated in this study, ovarian pedicle bupivacaine administration was associated with lower UMPS scores and higher SOD and CAT activities, whereas circulating pro‐inflammatory cytokine concentrations remained comparable between groups. These findings should be interpreted as early clinical and biochemical associations rather than evidence of definitive systemic anti‐inflammatory or antioxidant effects.

## 5 Limitations

Several limitations should be considered when interpreting these findings. The a priori power analysis was based on the primary pain outcome rather than on cytokine concentrations, so the study was not designed to detect small or moderate differences in IL‐1β, IL‐6 or TNF‐α. Given the marked inter‐individual variability of these mediators, the lack of significant between‐group differences may partly reflect a Type II error, and larger trials with cytokine‐specific power calculations would be needed to determine whether ovarian pedicle bupivacaine has a measurable effect on the systemic inflammatory response. The work was also carried out at a single centre by one surgical team. This kept the technique consistent and reduced procedural variability, but it inevitably narrows external validity, and the results may not transfer directly to other centres, instruments, perioperative protocols or surgeons with different laparoscopic experience. Because only ASA I dogs were enrolled, the findings apply to healthy animals undergoing elective LOE and should not be extended to geriatric, obese, or systemically compromised patients, in whom pain, inflammatory and antioxidant responses may behave differently. Although follicular‐phase animals were excluded on the basis of vaginal cytology, circulating sex hormones were not measured, so an effect of hormonal status on the inflammatory and antioxidant variables cannot be entirely excluded.

The short observation window is a further constraint. Pain scoring and blood sampling were confined to the first 4 h after surgery, which captures the early recovery period but not the full pain trajectory or the later rise in pro‐inflammatory cytokines that is typically seen between 6 and 24 h. The non‐significant cytokine results should therefore be read as the absence of an early, short‐term difference rather than as evidence that inflammation was unaffected. This window was chosen deliberately, since it preceded the first meloxicam dose and allowed the analgesic effect of bupivacaine to be assessed without NSAID interference; even so, meloxicam administered afterwards may have blunted later cytokine release and obscured subtle between‐group differences. The rescue threshold (UMPS > 10/27) was set a priori from published recommendations and comparable canine ovariectomy studies, but intervention cut‐offs differ across scales and reports; no dog reached this threshold and the highest individual score was 5/27, so the pain data should be read within these predefined criteria and the NSAID‐free 4‐h window. Pain was assessed with the UMPS alone, which, although validated, remains partly observer‐dependent, and adding objective measures such as serum cortisol, algometry or force‐plate analysis would have given a fuller picture of nociception and perioperative stress.

The biochemical assessment has its own constraints. Serum bupivacaine was not quantified, and given the volume required with the 0.5% solution, some systemic absorption cannot be ruled out and may have contributed to the changes seen in SOD and CAT. Both enzymes were measured in serum rather than in hemolyzed erythrocytes normalized to haemoglobin, so the present values reflect circulating antioxidant enzyme activity and are not directly comparable with erythrocyte‐based measurements. The redox evaluation was also restricted to these two enzymes, with no direct markers of oxidative damage (MDA, protein carbonyls, 8‐OHdG) or global oxidant status (TOS). For this reason, the higher SOD and CAT activities cannot, on their own, be equated with reduced oxidative stress, as they may equally represent a compensatory response to increased reactive oxygen species. A broader panel covering oxidant load, oxidative damage, and antioxidant capacity, combined with longer postoperative monitoring and objective nociceptive and endocrine measures, would help clarify both the mechanism and the clinical relevance of ovarian pedicle‐targeted analgesia in future multicentre studies.

## 6 Conclusion

This study showed that topical administration of bupivacaine HCl to the ovarian pedicle during LOE was associated with improved early postoperative analgesia in female dogs. In the BPLOVE group, higher serum SOD and CAT activities were observed during the early perioperative period, indicating a measurable change in antioxidant enzyme activity. These findings support the potential role of ovarian pedicle bupivacaine administration as part of multimodal analgesia for canine laparoscopic surgery, while the biochemical results should be interpreted specifically within the scope of antioxidant enzyme activity.

## Author Contributions

**Alper Yasin Çiplak**: conceptualization, data curation, formal analysis, funding acquisition, investigation, methodology, project administration, resources, software, supervision, validation, visualization, writing – original draft preparation, writing – review and editing. **Bülent Polat**: conceptualization, methodology, funding acquisition, project administration, resources, supervision, validation, writing – review and editing.

## Funding

This work was financially supported by the Scientific Research Projects Coordination Unit (BAP) of Atatürk University (Project No. 13014). The present manuscript was produced from the corresponding doctoral dissertation.

## Ethics Statement

This study was conducted following approval by the Atatürk University Animal Experiments Local Ethics Committee (Erzurum, Turkiye; Decision No: 2023/08) and was performed in accordance with institutional and national guidelines for the care and use of animals in research.

## Conflicts of Interest

The authors declare no conflicts of interest.

## Data Availability

The data supporting the findings of this study are available from the corresponding author upon reasonable request.
